# Red Cotton Stamen Extracts Mitigate Ferrous Sulfate-Induced Oxidative Stress and Enhance Quality in Bull Frozen Semen

**DOI:** 10.3390/vetsci12070674

**Published:** 2025-07-17

**Authors:** Jiraporn Laoung-on, Jakree Jitjumnong, Paiwan Sudwan, Nopparuj Outaitaveep, Sakaewan Ounjaijean, Kongsak Boonyapranai

**Affiliations:** 1 Research Institute for Health Sciences, Chiang Mai University, Chiang Mai 50200, Thailand; jiraporn.l@cmu.ac.th (J.L.-o.); nop.outaitaveep@gmail.com (N.O.); 2 Office of Research Administration, Chiang Mai University, Chiang Mai 50200, Thailand; 3 Department of Animal and Aquatic Sciences, Faculty of Agriculture, Chiang Mai University, Chiang Mai 50200, Thailand; jakree.j@cmu.ac.th; 4 Department of Anatomy, Faculty of Medicine, Chiang Mai University, Chiang Mai 50200, Thailand; paiwan.sudwan@cmu.ac.th

**Keywords:** ROS, *Bombax ceiba*, sperm motility, sperm viability, phytochemicals

## Abstract

Infertility is a significant global health concern, and incorporating antioxidants into sperm preparation media is one strategy to enhance sperm quality and decrease infertility rates. This study aimed to investigate the phytochemical compounds of red cotton stamen extracts and their effects as antioxidants in improving the quality of bull frozen semen. Among the extracts, RCU contained the highest levels of total phenolics, total tannins, and total monomeric anthocyanins along with the strongest ABTS free radical scavenging activity and protein denaturation inhibition. Exposing sperm to FeSO_4_-induced oxidative stress resulted in significantly reduced motility, viability, and normal morphology. However, treatment with RCD, RCU, and RCM improved these parameters. Additionally, the FeSO_4_-induced group showed elevated levels of reactive oxygen species (ROS) and advanced glycation end products (AGEs) compared to the normal control, whereas all red cotton stamen extracts effectively reduced these levels. In conclusion, red cotton stamen extracts, rich in phenolic bioactive compounds, demonstrated strong free radical scavenging capacity and improved sperm motility, viability, and morphology by neutralizing free radicals and enhancing antioxidant defenses. These findings suggest that the red cotton stamen extracts, particularly RCD and RCU, offer benefits for sperm preservation.

## Simple Summary

Our research investigated plant extracts, including RCD, RCU, and RCM, for their potential to scavenge free radicals induced by FeSO_4_. Sperm exposed to FeSO_4_-induced oxidative stress were treated with these extracts. Our data showed that red cotton stamen extracts, particularly RCD and RCU, exhibited the highest potential to improve sperm quality by neutralizing free radicals and enhancing antioxidant levels. These findings strengthen the evidence that natural plant-derived antioxidants can significantly improve sperm quality.

## 1. Introduction

Infertility is a significant issue that impacts global population growth rates, population structure, and economic conditions worldwide [1]. Male reproductive abnormalities contribute substantially to infertility and significantly affect population growth rates [2]. Various environmental and intrinsic factors can lead to male infertility through mechanisms involving oxidative stress [3]. While free radicals are produced at baseline levels and play essential roles in sperm capacitation, hyperactivation, and sperm-oocyte fusion, excessive production can overwhelm the antioxidant defense system, leading to sperm damage [4]. Cellular damage resulting from an imbalance between reactive oxygen species (ROS) and antioxidant defenses, along with reduced antioxidant capacity, is recognized as a significant contributor to male infertility [5]. Iron (II) sulfate is a crucial element in various chemical processes with significant implications in both industrial and biological contexts. Ferrous sulfate, one of the commonly used iron salts, can induce oxidative stress through the generation of superoxide radicals [6]. Sperm are particularly vulnerable to oxidative damage due to the high levels of polyunsaturated fatty acids in their plasma membranes and the relatively low levels of cytoplasmic antioxidant enzymes [3,7,8,9]. Oxidative stress from external factors such as temperature, stress, air pollution, and chemical contamination can cause abnormalities in spermatogenesis, sperm concentration, motility, viability, and morphology [3,10], all of which adversely affect male fertility [11]. Additionally, oxidative stress impairs male fertility by reducing sperm capacitation, acrosome reaction, hyperactivation, and sperm–oocyte binding [3]. The consumption of antioxidants is a critical factor in preventing oxidative stress and is relevant to the medical treatment of male infertility [3]. Additionally, incorporating antioxidants into the sperm preparation media is one of the treatment strategies used to enhance sperm quality and address increasing infertility [12,13]. While synthetic antioxidants are often convenient and effective, they are associated with higher costs and potential side effects, including carcinogenesis, fibrosis, and embryotoxicity [14]. Consequently, plants rich in phytochemical compounds represent a significant source of natural antioxidants and offer a viable alternative for oxidative protection [5,15].

Red cotton (*Bombax ceiba* L.: *B. ceiba*) is a spectacular flowering tree that belongs to the Bombacaceae family [16]. This plant is predominantly found in Africa, Australia, and Asia, particularly throughout northern Thailand, where it is locally known as “Ngui” [17]. Cotton stamens are regarded as an essential ingredient in traditional Lanna cuisine, represented by “Nam Ngiao,” a curry soup topped with rice noodles, making this plant culturally significant and popular in northern Thailand [17]. The cotton tree grows flowers in three varieties: red, orange, and occasionally white [18]. In Thailand, the red and orange varieties are present, with the orange stamen being more popular and economically valuable in the vegetable market due to its more appealing color compared to the red stamen. As a result of the high demand for orange stamens, the red stamens often become agricultural waste [17]. The cotton tree has been applied for treating multiple diseases, including antidiabetic, antioxidant, antibacterial, and anti-Hepatitis B viral properties [19,20,21]. Previous studies have shown that the orange stamens are rich in essential phytochemical compounds, particularly phenolic and flavonoid compounds, and have demonstrated the potential to enhance sperm quality [22].

Although orange cotton stamen has been reported to enhance sperm quality, red cotton stamen, often considered agricultural waste, has not been investigated or utilized for its potential to reduce oxidative stress or improve sperm quality. Therefore, this study aims to investigate the phytochemical compounds in red cotton stamen extracts and evaluate their effects on antioxidant capacity and quality of bull frozen sperm under FeSO_4_-induced oxidative stress.

## 2. Materials and Methods

### 2.1. Plant Collection and Extraction

Red cotton stamens were collected from an open field in the lower northern region of Thailand (Laplae district, Uttaradit Province, Thailand). The plant specimens were submitted to and verified by Herbarium, Faculty of Pharmacy, Chiang Mai University (fl. 002). The red cotton stamens were dried in the shade, pulverized, and extracted using three different methods according to the previous study [22]: decoction (red cotton decoction extract: RCD), ultrasonication (red cotton ultrasonicated extract: RCU), and microwave-assisted extraction (red cotton microwave-assisted extract: RCM). The plant extracts were filtered and evaluated for their phytochemical composition and antioxidant activity. Subsequently, the extracts were lyophilized and stored at −20 °C prior to use in antioxidant and sperm quality assessment.

### 2.2. Total Phenolic and Total Tannin Content Determination

The total phenolic and total tannin contents of RCD, RCU, and RCM at a concentration of 1 mg/mL were determined using the Folin–Ciocalteu assay. Gallic acid and tannic acid were used as standard calibrators for total phenolic and total tannin content, respectively. For the assay, 50 µL plant extract (1 mg/mL) was mixed with 250 µL of 10% Folin–Ciocalteu reagent, followed by the addition of 200 µL of 1 M Sodium carbonate (Na_2_CO_3_). The mixtures were incubated at room temperature for 30 min. Total phenolic content was measured at 765 nm, and total tannin content was measured at 700 nm using a microplate reader (BMG LABTECH, Ortenberg, Germany). The results were expressed as microgram equivalents per gram of dried plant weight.

### 2.3. Total Anthocyanins Determination

The total monomeric anthocyanin content of the plant samples was determined using the pH-differential method, according to the previous study [23]. Two separate dilutions were prepared using 0.025 M potassium chloride buffer at pH 1.0 and 0.4 M sodium acetate buffer at pH 4.5. A 100 µL aliquot of the sample solution was mixed with 900 µL of either the buffer pH 1.0 or pH 4.5 in a 5 mL tube. The mixtures were then incubated at room temperature for 15 min. The concentration of monomeric anthocyanins in the extracts was calculated using the following formula: Monomeric anthocyanin pigment (µg/mL) = (A × MW × DF × 1000)/(ε × 1), where anthocyanin content was expressed as cyanidin-3-glucoside, when MW = 449.2 and ε = 26,900.

### 2.4. Total Flavonoid Determination

The total flavonoid content was measured by mixing 50 µL of potassium acetate with 700 µL of distilled water, 50 µL of 10% aluminum chloride, and 100 µL of the extract solution in a test tube. The mixture was incubated at room temperature for 30 min, after which the absorbance was measured at 415 nm using a microplate reader. The total flavonoid content was expressed as micrograms of quercetin equivalents per gram of dried plant weight.

### 2.5. Lycopene Content Determination

The lycopene content of the plant samples was determined using a hexane–ethanol–acetone (2:1:1, *v*/*v*) mixture, according to a previous study [24]. Initially, 1 g of each dried sample was mixed with 1 mL of distilled water, vortexed, and incubated in a water bath at 30 °C for 60 min. Subsequently, 8 mL of a hexane–ethanol–acetone mixture (2:1:1) was added, followed by vortexing, and incubation in the dark for 10 min. Afterward, 1 mL of distilled water was added, and the mixture was vortexed again. The supernatants were collected and measured at 503 nm by a spectrophotometer. The lycopene concentration in the extract was calculated using the following formula: Lycopene (mg/g of dried plant weight) = A_503_ × 537 × 8 × 0.55/0.10 × 172.

### 2.6. High-Performance Liquid Chromatography (HPLC) Analysis of Phenolic Compounds

Identification and quantification of phenolic compounds related to sperm quality in red cotton stamen extracts were performed using reverse-phase high-performance liquid chromatography (HPLC). Analysis was conducted on a Shimadzu SIL-20AC Prominence Autosampler HPLC system (Shimadzu, Tokyo, Japan) equipped with a multi-wavelength detector, following the method described in a previous study [25]. The separation was carried out on a Purospher^®^ Star RP-18 (Agilent 1260 Infinity Binary LC, Santa Clara, CA, USA) endcapped column (150 × 4.60 mm, 5 µm particle size). The mobile phase consisted of solvent A (0.1% formic acid in water, 92%) and solvent B (acetonitrile, 8%). A 10 µL sample volume was injected for analysis. Detection was performed at a wavelength of 250 nm, and compounds were identified by comparing the retention times and UV-Vis spectral characteristics of the eluted peaks with those of reference standards. The retention times of gallic acid, chlorogenic acid, caffeic acid, and ferulic acid were 2.58, 6.82, 7.64, and 18.08, respectively.

### 2.7. Antioxidant Capacity Determination

#### 2.7.1. 2,2-Diphenyl-1-Picrylhydrazyl (DPPH) Radical Scavenging Assay

The DPPH radical scavenging assay was used to evaluate the free radical scavenging activity of red cotton stamen extracts. Fifty microliters (µL) of each extract at varying concentrations were added to 200 µL of a 0.004% DPPH solution in methanol. Gallic acid served as the positive control. The mixtures were incubated at room temperature for 30 min in the dark. Absorbance was measured at 515 nm using a microplate reader, and the results were used to determine the half-maximal inhibitory concentration (IC_50_).

#### 2.7.2. 2,2′-Azino-di-[3-Ethylbenzthiazoline Sulfonate] (ABTS) Radical Scavenging Assay

The free radical scavenging capacity of RCD, RCU, and RCM was evaluated using the ABTS assay. An ABTS stock solution (7 mM) was prepared with distilled water and stored at 4 °C in the dark. The solution was then diluted to achieve an absorbance of 0.7 at 734 nm. Subsequently, 50 µL of each concentration of red cotton stamen extract was added to 200 µL of ABTS working solution and incubated at room temperature in the dark for 30 min. Absorbance was measured at 515 nm using a microplate reader, with gallic acid serving as the positive control. The IC_50_ was calculated for each extract.

### 2.8. Inhibition of Protein Denaturation Assay

Reaction mixtures were incubated at 37 °C for 15 min, followed by heating at 70 °C for 5 min. After cooling to room temperature, the absorbance was measured before and after protein denaturation for each concentration at 600 nm using a microplate reader, with diclofenac serving as the positive control [26]. The IC_50_ was subsequently calculated.

### 2.9. Experimental Design

**Figure 1:**
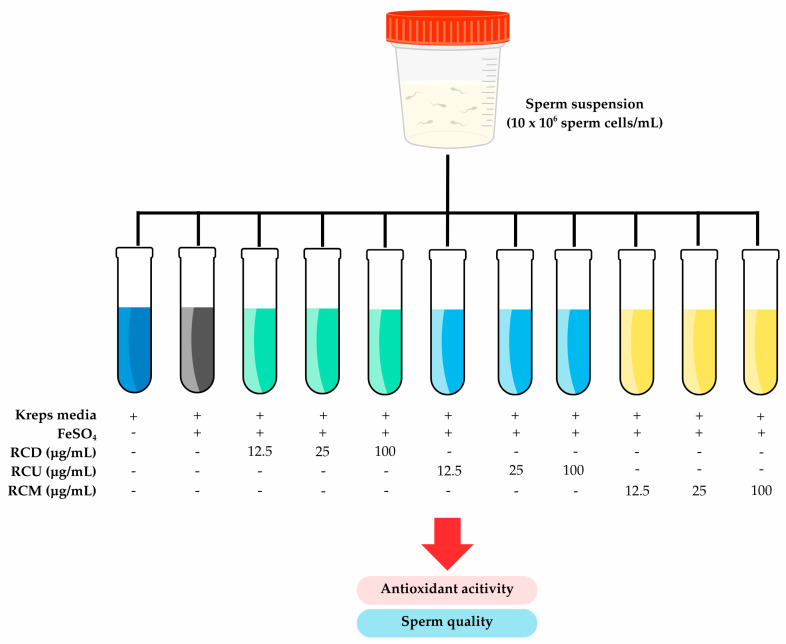
A schematic diagram showing the experimental design. The different tube colors were demonstrated the different extraction methods.

Frozen bull semen was purchased from Namchuea Wongwi Company Ltd. (Bangkok, Thailand). After thawing, the semen was washed twice with Krebs solution. The sperm viability was evaluated, yielding a viability rate of 95.67 ± 1.86%. The semen sample was prepared in Krebs medium at a concentration of 10 × 10^6^ sperm/mL for use in the antioxidant activity study of plant extracts on a sperm cell model. The sperm suspension was divided into 11 groups as follows: Group I (control): sperm treated with Krebs solution; Group II (oxidative induction): sperm treated with 20 µg/mL ferrous sulfate (Fe); Group III–V: sperm treated with RCD at doses of 6.25, 25, and 100 µg/mL, respectively; Group VI–VIII: sperm treated with RCU at doses of 6.25, 25, and 100 µg/mL, respectively; and Group IX–XI: sperm treated with RCM at doses of 6.25, 25, and 100 µg/mL, respectively. For each treatment, 500 µL of the sperm suspension was placed in a test tube and incubated at 37 °C for 3 h to mimic physiological conditions and allow for adequate interaction between the antioxidant compounds and sperm cells, as recommended in previous studies evaluating oxidative stress and antioxidant activity in sperm [27]. The experiment was conducted in triplicate. After incubation, the samples were centrifuged at 2500 rpm for 5 min to separate the supernatant from the sperm pellet. The diagram of the experimental design is presented in Figure 1.

### 2.10. Determination of Sperm Reactive Oxygen Species (ROS) Production

The antioxidant effects of RCD, RCU, and RCM on ROS production in sperm exposed to Fe-induced oxidative stress were assessed using the DCFH-DA (2′,7′-dichlorodihydrofluorescein diacetate) assay [28]. Following incubation, 100 µL of sperm solution from each treatment group was transferred into a 96-well plate. Then, 10 µL of 20 µM DCFH-DA was added to each well, and the plate was incubated at 37 °C with 5% CO_2_ for 30 min in the dark. ROS production was quantified by measuring fluorescence intensity at an excitation wavelength of 488 nm and an emission wavelength of 617 nm using a microplate reader.

### 2.11. Determination of Inhibition of Advanced Glycation End Products (AGEs) Formation

One hundred microliters of supernatant from each sample was transferred to a 96-well plate to assess the formation of advanced glycation end products (AGEs) using a microplate reader (excitation at 360 nm, emission at 460 nm) [29]. A standard calibration curve was established using quinine hemisulfate, and the results were expressed in mEq/µmol quinine hemisulfate per liter.

### 2.12. Sperm Qualities Evaluation

#### 2.12.1. Determination of Sperm Motility

Sperm motility characteristics, including non-motile sperm, progressive motility, circular motility, and non-progressive motility, were analyzed in triplicate for each sample using video recordings under a light microscope (Olympus CX31, Olympus Corporation, Tokyo, Japan) at 400× magnification. Two observers, blinded to the treatment groups, performed the analyses. This method is practical and essential for rapid, real-time data collection, enabling simultaneous assessment of multiple parameters and showing good correlation with a sperm motility analysis system [30,31]. A total of 200 sperm cells were counted and classified for each sample to ensure statistical validity [31].

#### 2.12.2. Determination of Sperm Viability

Sperm viability was assessed using a DAPI/PI fluorometric assay. A 100 µL aliquot of sperm solution from each treatment group was transferred into a 96-well plate. The samples were stained with 5 µL of propidium iodide (PI) solution (5 µg/mL), and incubated at 37 °C for 15 min, followed by the addition of 10 µL of 4′,6-diamidino-2-phenylindole dihydrochloride (DAPI) solution (1 µg/mL) [25]. Fluorescence intensity was measured using a microplate reader (PerkinElmer, Singapore) at excitation wavelengths of 360 nm and 530 nm, and emission wavelengths of 460 nm and 620 nm.

#### 2.12.3. Classification of Sperm Morphology

Microscopic photographs of sperm stained with TB/Giemsa and observed under a light microscope at 1000× magnification were used for morphological assessment. For each treatment, a total of 100 sperm cells were evaluated and classified according to morphological criteria [32].

### 2.13. Statistical Analysis

Experimental data are presented as mean ± standard deviation (SD). The Kolmogorov–Smirnov test was used to assess normality. For cell-free systems, one-way ANOVA followed by Duncan’s post hoc test was used to compare group means for phytochemical concentrations and antioxidant activities. Depending on the data distribution, either an independent *t*-test or Mann–Whitney U test was applied to compare treatment groups with the MZ and control groups. For nonparametric comparisons among multiple groups, the Kruskal–Wallis test followed by the Mann–Whitney U test was used. All experiments were performed in triplicate, and statistical significance was set at *p* < 0.05.

## 3. Results

### 3.1. Identification and Quantification of Phytochemical Compounds

**Figure 2:**
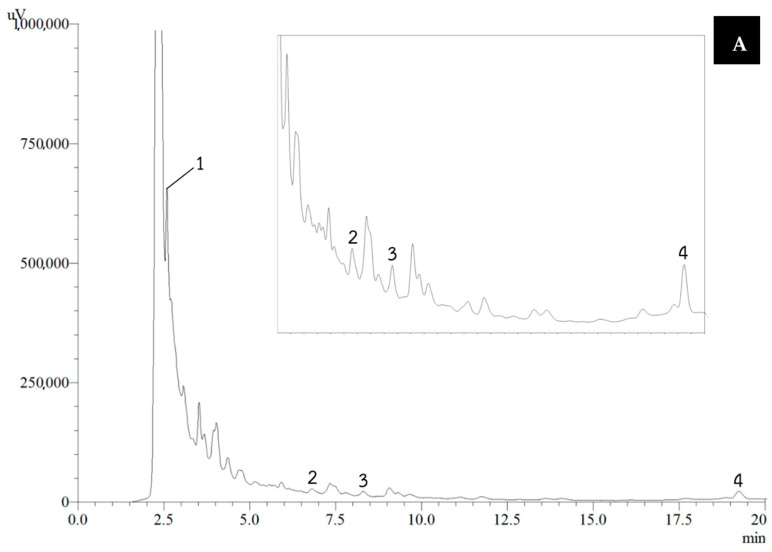

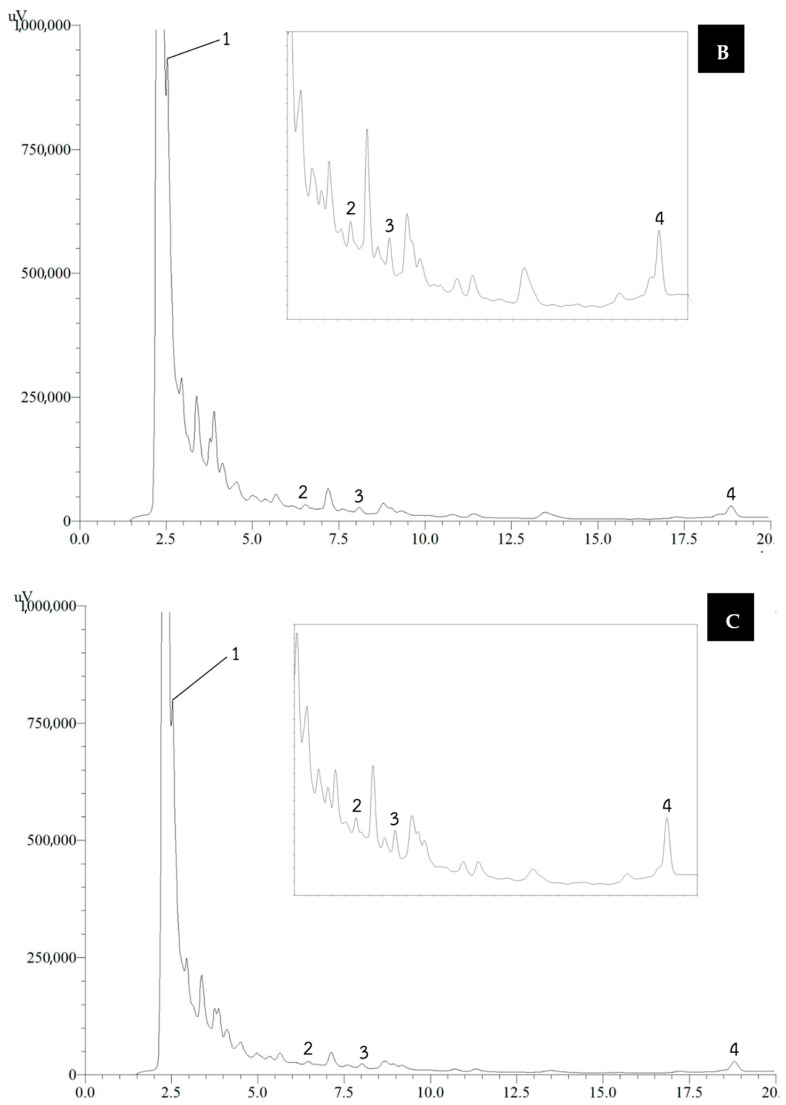
HPLC chromatograms of phenolic acids shown in the RCD (**A**), the RCU (**B**), and the RCM (**C**) from Column, Purospher^®^ Star PR-18; mobile phase, 0.1% formic acid in water and 8% acetonitrile; flow rate, 0.8 mL/min; detection wavelength, 250 nm. Peak identification: peak 1, gallic acid; peak 2, chlorogenic acid; peak 3, caffeic acid; and peak 4, ferulic acid.

Red cotton stamens were extracted using three methods: decoction (RCD), ultrasonication (RCU), and microwave-assisted extraction (RCM). RCU exhibited significantly higher total phenolic and total tannin contents compared to RCM, while no significant difference was observed between RCU and RCD. Moreover, RCU showed the highest total monomeric anthocyanin content, which was significantly greater than that of both RCD and RCM. However, no significant differences among the extraction methods were observed in percentage yield, total flavonoid concentration, or lycopene content (Table 1). Representative HPLC chromatograms of RCD, RCU, and RCM are shown in Figure 2. Concentrations were determined by comparing the peak areas of the sample components to those of authentic standards with known concentrations, with the results summarized in Table 2. RCU exhibited higher ferulic acid content compared to RCD and RCM. However, no significant differences were observed among the extraction methods for gallic acid, chlorogenic acid, and caffeic acid levels.

### 3.2. Antioxidant Capacities and Inhibition of Protein Denaturation in Cell-Free System

**Figure 3:**
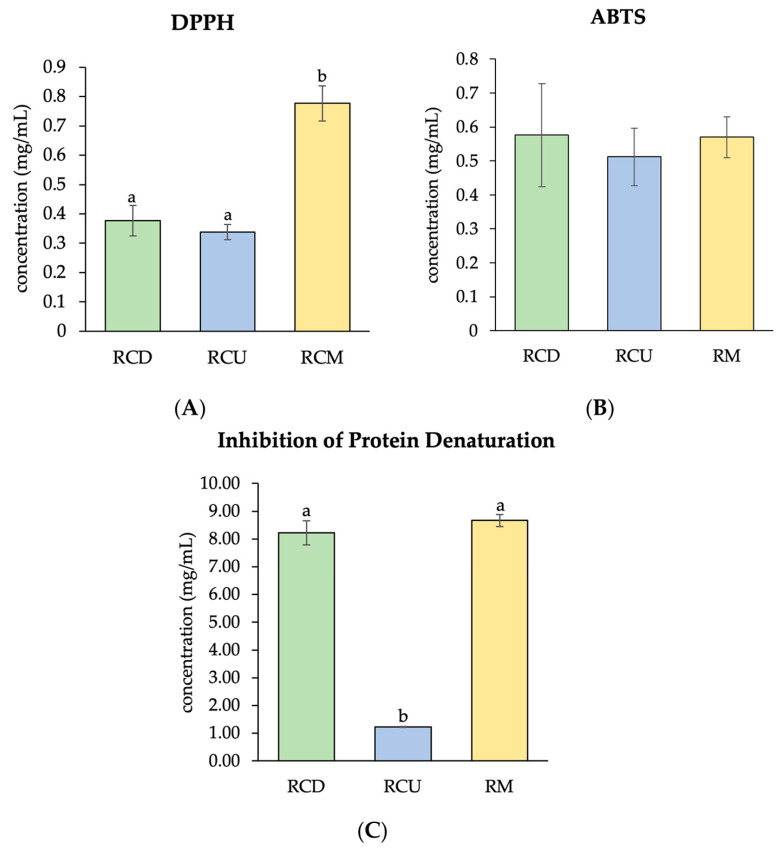
The half-maximal inhibitory concentration of DPPH (**A**), ABTS (**B**), and inhibition of protein denaturation (**C**) of RCD, RCU, and RCM is presented as mean value ± SD (error bars). Data were collected from three replications (*n* = 3). ^a,b^ Different letters indicate significant differences between groups at *p* < 0.05.

The half-maximal inhibitory concentration (IC_50_) for 2,2-diphenyl-1-picrylhydrazyl (DPPH) radical scavenging was significantly lower for RCD and RCU compared to RCM (Figure 3A). In contrast, the IC_50_ values for ABTS radical scavenging did not differ significantly among the extraction methods (Figure 3B). Additionally, RCU showed a significantly lower IC_50_ value for inhibition of protein denaturation compared to RCD and RCM (Figure 3C).

### 3.3. Sperm Reactive Oxygen Species (ROS)

**Figure 4:**
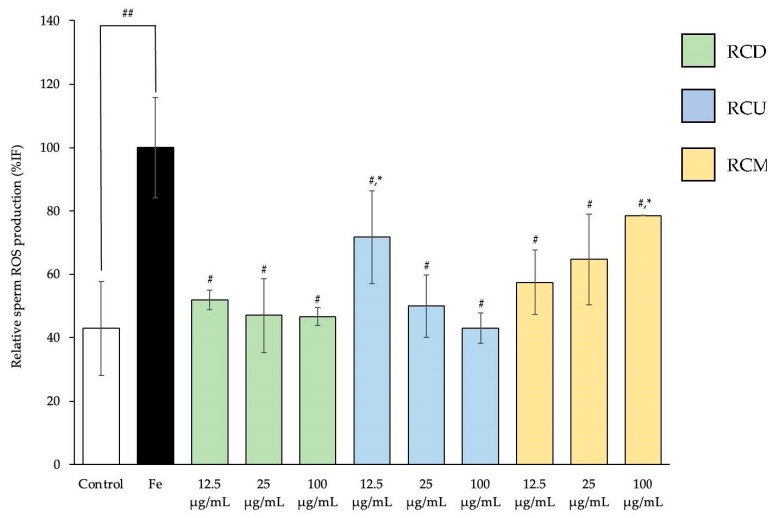
Mean value ± standard deviation (error bars) of relative sperm ROS production in bull frozen sperm sample treated under the following conditions: control; Fe; Fe with RCD, RCU, and RCM at the dose of 12.5, 25, and 100 µg/mL (Fe: Ferrous sulfate, RCD: red cotton decoction extraction, RCU: red cotton ultrasonicated extraction, and RCM: red cotton microwave-assisted extraction). Data were obtained from three replications (n = 3). * Demonstrates statistically significant deviations from the control group. ^#^ Indicates a statistically significant difference compared to the Fe-treated group. ^##^ The significance levels at which the Fe-treated group differ significantly from the control group (*p* < 0.05). %IF (fluorescence intensity) represents the percentage of fluorescence intensity relative to a FeSO_4_-induced oxidative stress, reflecting the degree of ROS activity in sperm cells.

The relative ROS production in bull frozen sperm treated with various extracts under Fe-induced oxidative stress is presented in Figure 4. The Fe-treated group exhibited a significant increase in ROS production compared to the control group. Treatment with RCD, RCU, and RCM significantly reduced ROS levels relative to the Fe-treated group. Notably, RCD at all doses, RCU at 25 and 100 µg/mL, and RCM at 12.5 and 25 µg/mL effectively suppressed ROS production to levels comparable to the normal control group.

### 3.4. Inhibition of Advanced Glycation End Products (AGEs)

**Figure 5:**
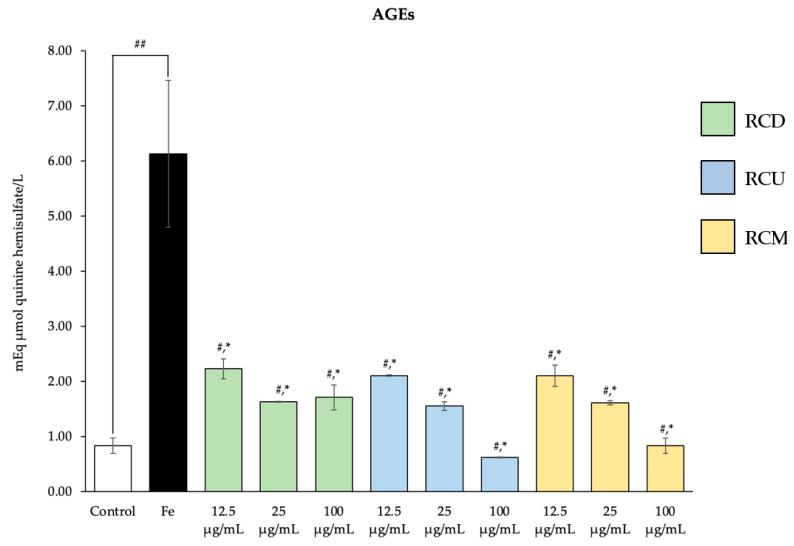
Mean value ± standard deviation (error bars) of AGEs of the bull frozen sperm sample treated under the following conditions: control; Fe; Fe with RCD, RCU, and RCM at the dose of 12.5, 25, and 100 µg/mL (Fe: Ferrous sulfate, RCD: red cotton decoction extraction, RCU: red cotton ultrasonicated extraction, and RCM: red cotton microwave-assisted extraction). Data were obtained from three replications (n = 3). * Indicates statistically significant deviations from the control group (*p* < 0.05). ^#^ Indicates a significant difference compared to the Fe-treated group (*p* < 0.05). ^##^ Indicates the significance levels at which the Fe-treated group differs significantly from the control group (*p* < 0.05).

Sperm treated with Fe showed a significantly higher level of AGE formation compared to the control group. Treatments with red cotton stamen extracts significantly reduced AGE levels relative to the Fe-treated group, although the levels remained elevated compared to the control group (Figure 5).

### 3.5. Sperm Quality

#### 3.5.1. Sperm Motility

The percentage of non-motile sperm increased significantly in the Fe-treated group compared to the normal control group. However, co-treatment with Fe and any concentration of RCD, RCU, or RCM resulted in a notable reduction in non-motility compared to the Fe-only group. Specifically, sperm treated with Fe and RCU at 25 and 100 µg/mL showed a significant decrease in non-motility compared to both the control and Fe groups. Progressive and non-progressive sperm motility were significantly reduced in the Fe group compared to the control group. However, co-treatment with Fe and RCD, RCU, or RCM led to marked improvements in both types of motility relative to the Fe group. Notably, RCU at 25 and 100 µg/mL significantly increased progressive motility compared to both the control and Fe groups (Table 3).

#### 3.5.2. Sperm Viability

**Figure 6:**
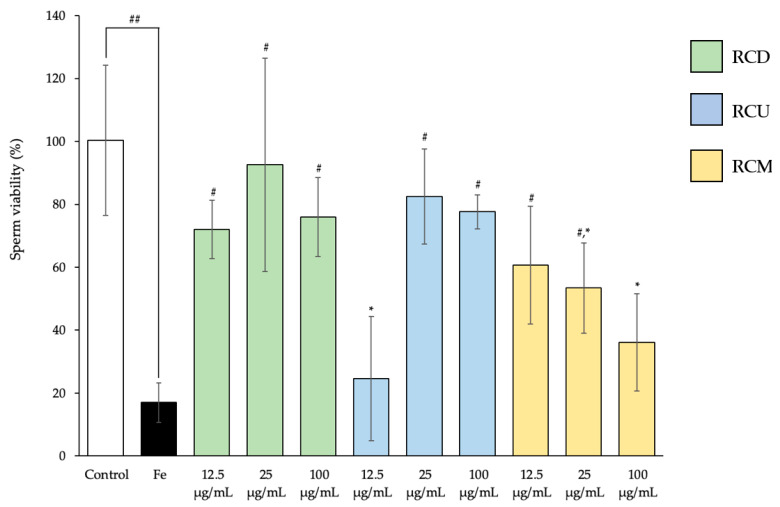
The percentage of viable bull sperm under the following treatment conditions: control; Fe; Fe with RCD, RCU, and RCM at the dose of 12.5, 25, and 100 µg/mL (Fe: Ferrous sulfate, RCD: red cotton decoction extraction, RCU: red cotton ultrasonicated extraction, and RCM: red cotton microwave-assisted extraction). The data are presented as mean value ± SD (error bars). Data were obtained from three replications (n = 3). * Indicates statistically significant deviations from the control group (*p* < 0.05). ^#^ Indicates the significant differences compared to the Fe-treated group (*p* < 0.05). ^##^ The significance levels at which the Fe-treated group differ significantly from the control group (*p* < 0.05).

The percentage of viable sperm was significantly reduced in the Fe-treated group compared to the control group. However, co-treatment with Fe and RCD (all doses), RCU (25 and 100 µg/mL), or RCM (12.5 and 25 µg/mL) significantly increased sperm viability compared to the Fe-only group. Notably, sperm treated with RCD (all doses), RCU (25 and 100 µg/mL), and RCM (12.5 µg/mL) showed viability levels comparable to those of the normal control group (Figure 6).

#### 3.5.3. Sperm Morphology Classification

**Figure 7:**
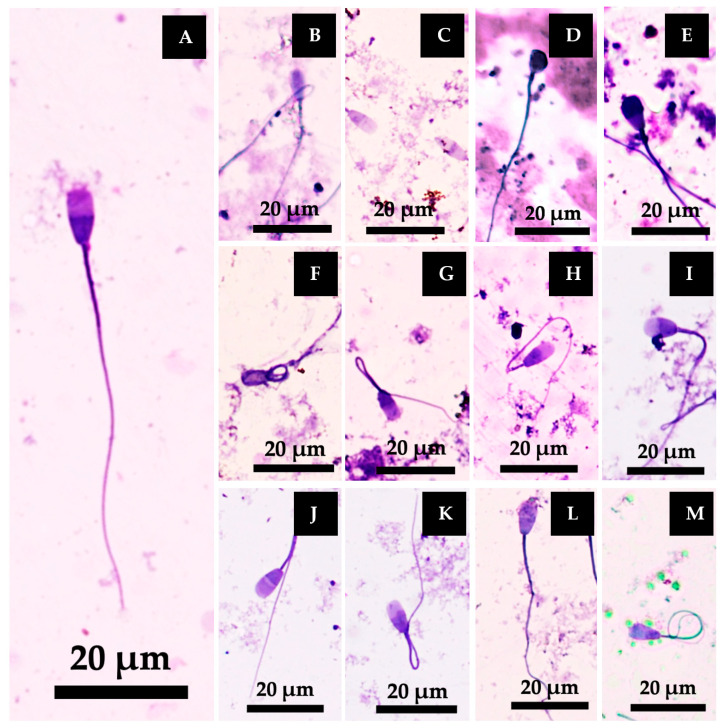
Morphological pattern of bull spermatozoa stained with TB/Giemsa observed at 400× magnification. (**A**) Normal sperm (**A**). (**B**–**E**) Sperm exhibiting various abnormal head morphologies: Small-shaped head (**B**), tapered head (**C**), irregular-shaped head (**D**), and pear-shaped head (**E**). (**F**–**I**) Sperm displaying combined head and tail abnormalities: tapered head with coiled tail (**F**), tapered head with hairpin (**G**), tapered head with bent tail (**H**), and macrocephalic head with edematous tail (**I**). (**J**–**M**) Sperm with different tail abnormalities: bent tail (**J**), hairpin tail (**K**), irregular-shaped tail (**L**), and coiled tail (**M**).

The pattern of sperm morphology is presented in Figure 7. The number of abnormal heads and tails was significantly higher in the Fe-treated group compared to the control group. However, treatment with all doses of RCD, RCU, and RCM significantly reduced the incidence of abnormalities relative to both the control and Fe-treated groups. RCD at 12.5 µg/mL and RCU at 25 µg/mL significantly reduced the number of abnormal tails compared to both the control and Fe groups. Additionally, the number of normal sperm was significantly increased in the RCD (12.5 µg/mL), RCU (all doses), and RCM (12.5 and 100 µg/mL) groups compared to the Fe-treated group (Table 4).

## 4. Discussion

In the present study, red cotton stamens were extracted using three methods: decoction, ultrasonic extraction, and microwave-assisted extraction. RCU contained the highest levels of total phenolics, total tannins, and total monomeric anthocyanins, followed by RCD and RCM, respectively. Similarly, a previous report showed that orange *Bombax ceiba* stamens extracted using ultrasonic extraction had the highest concentrations of total phenolics, total tannins, and total monomeric anthocyanins [22]. Ultrasonic extraction is a modern technique that uses ultrasound energy in combination with solvents to efficiently extract target compounds from natural products [33]. This method enhances solvent movement and penetration, resulting in reduced extraction time, lower energy consumption, and decreased extraction temperatures [34]. Polyphenols constitute a major class of phytochemicals that are soluble in organic solvents, with optimal extraction typically achieved at temperatures between 40 °C and 60 °C, while degradation may occur at temperatures exceeding 60 °C [35,36,37]. Similarly, total monomeric anthocyanin concentrations peaked between 20 °C and 60 °C, with heat-induced degradation occurring at higher temperatures [35,38]. Therefore, the high levels of total phenolics, total tannins, and total monomeric anthocyanins observed in the RCU are likely attributable to the use of ultrasound-assisted extraction at optimal temperature and duration, which enhances solvent penetration and compound solubility [39]. In contrast, the lower levels of these compounds in RCD and RCM may be due to degradation associated with the temperature and extraction methods employed, leading to the breakdown of these phytochemicals.

The HPLC chromatogram of red cotton stamen extracts revealed the presence of gallic acid, chlorogenic acid, caffeic acid, and ferulic acid. RCU exhibited a higher concentration of ferulic acid compared to RCD and RCM. Ferulic acid, a naturally occurring phenolic compound abundant in various plants, functions as a potent antioxidant [40], helping to prevent cellular damage [41]. Previous studies have demonstrated that ferulic acid contributes to the preservation of sperm quality in roosters [42], boars [43], rams [44], and goats [45]. Additionally, this study found that RCD and RCU demonstrated superior efficacy in ABTS radical scavenging compared to RCM. The ABTS assay employs a hydrogen donor to facilitate radical scavenging and antioxidant reactions [46]. Furthermore, RCU demonstrated higher efficacy in inhibiting egg protein denaturation than RCD and RCM. The egg albumin degeneration method serves as an alternative approach for evaluating the anti-inflammatory efficacy of herbal medication [26]. Overall, the findings indicate that red cotton stamen extracted via ultrasonication is an effective method for polyphenol extraction, exhibiting superior antioxidant and anti-inflammatory properties.

The current study found that within 3 h, induction of oxidative stress in bull sperm using FeSO_4_ resulted in increased free radical production. The study showed that sperm treated with FeSO_4_ exhibited higher levels of ROS and AGEs compared to the control group. Although low levels of ROS are essential for sperm to acquire fertilization capacity, excessive ROS can induce oxidative stress, impair sperm motility, viability, and DNA integrity, and potentially contribute to male infertility [47]. FeSO_4_ can generate ROS in sperm, leading to oxidative stress and impaired sperm function through the Fenton reaction, in which iron acts as a catalyst to produce highly reactive hydroxyl radicals from hydrogen peroxide and superoxide [8,48]. The resulting oxidative stress can damage sperm membranes, DNA, and proteins, thereby affecting sperm motility and fertilization potential, and may also impact the health of the offspring [5]. Nevertheless, the addition of red cotton stamen extracts, from all extraction methods, demonstrated a decrease in the levels of ROS and AGEs compared to sperm incubated only with FeSO_4_. These extracts may have positively influenced free radical scavenging under Fe-induced oxidative stress in bovine sperm, thereby helping to prevent cellular damage [7]. The incubation with FeSO_4_ for 3 h induced oxidative stress, leading to increased concentrations of ROS and AGEs. This oxidative stress from FeSO_4_ treatment reduced sperm motility, viability, and percentage of morphologically normal sperm cells. These findings are consistent with previous studies reporting that Fe-induced oxidative stress decreases sperm viability in both rat and cattle sperm in in vitro experiments [22,25]. However, in this study, the addition of RCD, RCU, and RCM reduced the negative effects of oxidative stress, specifically RCD and RCU. Similarly, the previous studies using orange *B. ceiba* stamen [22] and white *Nelumbo nuceifera* (*N. nuceifera*) petal extracts [25] showed enhanced sperm viability during the oxidative stress process induced by FeSO_4_. The results indicated that RCD and RCU contain bioactive components that may directly contribute to the scavenging of free radicals in Fe-induced oxidative stress in beef cattle sperm, providing a protective effect against cellular damage. Moreover, RCD and RCU exhibited the greatest capacity to improve sperm quality, indicating that these extracts are rich in essential bioactive compounds that may directly contribute to free radical scavenging in beef cattle sperm, thereby reducing ROS and AGEs. These results suggest that red cotton stamen may improve sperm quality under in vitro conditions and may have the potential to support reproductive health, particularly in human and animal models.

## 5. Conclusions

In conclusion, red cotton stamen extracts, particularly RCD and RCU, contain phenolic bioactive compounds and demonstrate strong potential for free radical scavenging in bull frozen sperm subjected to FeSO_4_-induced oxidative stress. The addition of these extracts to the sperm media improved sperm motility, viability, and normal morphology by neutralizing free radicals and enhancing antioxidant levels. The findings suggest that red cotton stamen extracts, especially RCD and RCU, offer promising benefits for sperm preservation. Future studies should evaluate the safety, efficacy, and mechanisms of red cotton stamen extracts in improving sperm quality under oxidative stress and explore their application in sperm preservation for assisted reproductive technologies.

## Figures and Tables

**Table 1:** Percentage yield, total phenolic, total tannins, total monomeric anthocyanins, total flavonoid, and lycopene content of red cotton stamens at different methods of extraction.

Sample	Yield (%)	Total Phenolic (µg GAE/g Dried Weight)	Total Tannin (µg TAE/g Dried Weight)	Total Monomeric Anthocyanins (µg Cyanidin-3-Glucoside E/g Dried Weight)	Total Flavonoid (µg QE/g Dried Weight)	Lycopene Content (×10^2^ µg/g Dried Weight)
RCD	21.81 ± 6.27	5.55 ± 0.27 ^b,c^	4.77 ± 0.23 ^b,c^	6.85 ± 1.04 ^a,b^	0.11 ± 0.06	41.71 ± 8.89
RCU	22.21 ± 4.90	5.84 ± 0.21 ^c^	4.98 ± 0.19 ^c^	7.99 ± 0.26 ^c^	0.13 ± 0.03	56.91 ± 28.90
RCM	21.19 ± 6.60	5.19 ± 0.21 ^a,b^	4.45 ± 0.17 ^a,b^	6.46 ± 0.26 ^a^	0.15 ± 0.01	40.30 ± 13.96

The results are presented as the mean value ± standard deviation (SD). All experiments were performed in triplicate, with three independent repetitions. ^a,b,c^ Different letters indicate significant differences between groups in the column data at *p* < 0.05. All parameters were analyzed by one-way ANOVA followed by Duncan’s test.

**Table 2:** The values of gallic acid, chlorogenic acid, caffeic acid, and ferulic acid of red cotton stamens at different methods of extraction.

Sample	µg/Mg Plant Extract			
	Gallic Acid	Chlorogenic Acid	Caffeic Acid	Ferulic Acid
RCD	0.61 ± 0.05	0.02 ± 0.01	0.02 ± 0.01	0.02 ± 0.01 ^a,b^
RCU	0.63 ± 0.06	0.02 ± 0.01	0.02 ± 0.01	0.03 ± 0.01 ^a^
RCM	0.65 ± 0.05	0.02 ± 0.01	0.02 ± 0.01	0.01 ± 0.01 ^b^

The results are presented as the mean value ± standard deviation (SD). All experiments were performed in triplicate, with three independent repetitions. ^a,b^ Different letters indicate significant differences between groups in the column data at *p* < 0.05. All parameters were analyzed by one-way ANOVA followed by Duncan’s test.

**Table 3:** Percentage of sperm motility and non-motility of bull frozen sperm sample treated with ferrous sulfate (Fe), RCD, RCU, RCM, and the control group.

Group	Concentration (µg/mL)	Percentage of Sperm Motility			Percentage of Sperm Non-Motility
		Progressive	Circle	Non-Progressive	
Control		1.67 ± 0.27	1.67 ± 1.04	15.83 ± 0.29	80.83 ± 0.76
Ferrous sulfate (Fe)	20 µg/mL	0.00 ± 0.00 ^##^	0.00 ± 0.00 ^##^	0.17 ± 0.29 ^##^	99.83 ± 0.29 ^##^
RCD	12.5 µg/mL	2.66 ± 1.60 ^#^	0.00 ± 0.00 *	21.50 ± 1.00 *^,#^	75.84 ± 1.04 *^,#^
	25 µg/mL	2.34 ± 1.04 ^#^	0.00 ± 0.00 *	22.33 ± 8.81 *^,#^	75.33 ± 9.83 ^#^
	100 µg/mL	0.84 ± 0.29 *^,#^	0.00 ± 0.00 *	13.33 ± 2.84 ^#^	81.83 ± 3.06 ^#^
RCU	12.5 µg/mL	2.17 ± 0.29 ^#^	0.00 ± 0.00 *	13.00 ± 3.28 ^#^	84.84 ± 3.55 ^#^
	25 µg/mL	5.00 ± 1.50 *^,#^	0.00 ± 0.00 *	44.17 ± 16.65 *^,#^	50.83 ± 18.11 *^,#^
	100 µg/mL	3.66 ± 1.26 *^,#^	0.00 ± 0.00 *	44.34 ± 10.50 *^,#^	52.00 ± 11.76 *^,#^
RCM	12.5 µg/mL	0.16 ± 0.29 *	0.00 ± 0.00 *	12.84 ± 0.58 *^,#^	87.00 ± 0.50 *^,#^
	25 µg/mL	0.00 ± 0.00 *	0.00 ± 0.00 *	9.17 ± 0.77 *^,#^	90.83 ± 0.77 *^,#^
	100 µg/mL	0.50 ± 0.00 *^,#^	0.00 ± 0.00 *	12.67 ± 2.26 *^,#^	86.83 ± 2.25 *^,#^

A Kruskal–Wallis test and a Mann–Whitney U test were used to analyze the percentages of motility and non-motility of the sperm. Nine replicates (n = 3) were used to collect the data. * Demonstrates statistically significant deviations from the control group. ^#^ Indicates the significant differences compared to the Fe group. ^##^ The significance levels at which the Fe group differs significantly from the control group (*p* < 0.05).

**Table 4:** Number of the normal and abnormal sperm of beef cattle sperm sample treated with ferrous sulfate (Fe), RCD, RCU, RCM, and the control group.

Group	Concentration (µg/mL)	Number of Normal Sperm	Number of Abnormal Sperm		
			Head Only	Head and Tail	Tail Only
Control		45.33 ± 5.51	2.67 ± 0.58	6.33 ± 1.53	45.67 ± 3.51
Ferrous sulfate (Fe)	20 µg/mL	36.00 ± 6.00	6.00 ± 4.00	17.67 ± 6.51 ^##^	40.33 ± 4.51
RCD	12.5 µg/mL	69.33 ± 0.58 *^,#^	0.67 ± 0.58 *^,#^	0.00 ± 0.00 *^,#^	30.00 ± 0.01 *^,#^
	25 µg/mL	51.67 ± 13.50	0.67 ± 0.58 *^,#^	3.33 ± 0.58 *^,#^	44.33 ± 12.50
	100 µg/mL	51.67 ± 13.50	0.67 ± 0.58 *^,#^	1.67 ± 2.08 *^,#^	46.00 ± 11.00
RCU	12.5 µg/mL	58.33 ± 6.51 *^,#^	1.33 ± 1.15	1.33 ± 0.58 *^,#^	39.00 ± 8.00
	25 µg/mL	64.67 ± 0.58 *^,#^	0.67 ± 0.58 *^,#^	0.33 ± 0.58 *^,#^	34.33 ± 0.58 *^,#^
	100 µg/mL	61.67 ± 3.51 *^,#^	0.00 ± 0.00 *^,#^	1.33 ± 1.53 *^,#^	37.00 ± 2.00 *
RCM	12.5 µg/mL	51.67 ± 1.53 ^#^	0.67 ± 0.58 *^,#^	1.67 ± 1.15 *^,#^	46.00 ± 1.00
	25 µg/mL	57.67 ± 19.50	1.67 ± 1.53	1.67 ± 1.53 *^,#^	39.00 ± 16.52
	100 µg/mL	52.00 ± 7.00 ^#^	0.00 ± 0.00 *^,#^	1.00 ± 1.00 *^,#^	47.00 ± 8.00

A Kruskal–Wallis test and a Mann–Whitney U test were used to analyze the number of the normal and abnormal sperm. Nine replicates (n = 3) were used to collect the data. * Demonstrates statistically significant deviations from the control group. ^#^ Indicates the significant differences compared to the Fe group. ^##^ The significance levels at which the Fe group differs significantly from the control group (*p* < 0.05).

## Data Availability

The original contributions presented in this study are included in the article. Further inquiries can be directed at the corresponding author.
